# Genomic characterization of explant tumorgraft models derived from fresh patient tumor tissue

**DOI:** 10.1186/1479-5876-10-125

**Published:** 2012-06-18

**Authors:** David J Monsma, Noel R Monks, David M Cherba, Dawna Dylewski, Emily Eugster, Hailey Jahn, Sujata Srikanth, Stephanie B Scott, Patrick J Richardson, Robin E Everts, Aleksandr Ishkin, Yuri Nikolsky, James H Resau, Robert Sigler, Brian J Nickoloff, Craig P Webb

**Affiliations:** 1 Laboratory for Translational Medicine, Van Andel Research Institute, Grand Rapids, MI, USA; 2 Sequenom Inc, San Diego, CA, USA; 3 GeneGo-Thomson Reuters, St. Joseph, MI, USA; 4 Program in Biospecimen Science, Van Andel Research Institute, Grand Rapids, MI, USA; 5 Research Essential Services, LLC, Plymouth, MI, USA; 6 Laboratory of Cutaneous Oncology, Van Andel Research Institute, Grand Rapids, MI, USA

**Keywords:** Tumorgrafts, Genomics, Translational models

## Abstract

**Background:**

There is resurgence within drug and biomarker development communities for the use of primary tumorgraft models as improved predictors of patient tumor response to novel therapeutic strategies. Despite perceived advantages over cell line derived xenograft models, there is limited data comparing the genotype and phenotype of tumorgrafts to the donor patient tumor, limiting the determination of molecular relevance of the tumorgraft model. This report directly compares the genomic characteristics of patient tumors and the derived tumorgraft models, including gene expression, and oncogenic mutation status.

**Methods:**

Fresh tumor tissues from 182 cancer patients were implanted subcutaneously into immune-compromised mice for the development of primary patient tumorgraft models. Histological assessment was performed on both patient tumors and the resulting tumorgraft models. Somatic mutations in key oncogenes and gene expression levels of resulting tumorgrafts were compared to the matched patient tumors using the OncoCarta (Sequenom, San Diego, CA) and human gene microarray (Affymetrix, Santa Clara, CA) platforms respectively. The genomic stability of the established tumorgrafts was assessed across serial *in vivo* generations in a representative subset of models. The genomes of patient tumors that formed tumorgrafts were compared to those that did not to identify the possible molecular basis to successful engraftment or rejection.

**Results:**

Fresh tumor tissues from 182 cancer patients were implanted into immune-compromised mice with forty-nine tumorgraft models that have been successfully established, exhibiting strong histological and genomic fidelity to the originating patient tumors. Comparison of the transcriptomes and oncogenic mutations between the tumorgrafts and the matched patient tumors were found to be stable across four tumorgraft generations. Not only did the various tumors retain the differentiation pattern, but supporting stromal elements were preserved. Those genes down-regulated specifically in tumorgrafts were enriched in biological pathways involved in host immune response, consistent with the immune deficiency status of the host. Patient tumors that successfully formed tumorgrafts were enriched for cell signaling, cell cycle, and cytoskeleton pathways and exhibited evidence of reduced immunogenicity.

**Conclusions:**

The preservation of the patient’s tumor genomic profile and tumor microenvironment supports the view that primary patient tumorgrafts provide a relevant model to support the translation of new therapeutic strategies and personalized medicine approaches in oncology.

## Background

Although direct transfer xenografts of fresh human tumors, or primary “tumorgrafts”, have been reported as early as the 1970s for testing of new pharmaceutical agents [1-4], only recently has there been a resurgence of interest in this alternative to the more traditional cell line xenograft models. This has been due, in part, to the growing realization that drugs which work in the traditional cell line xenograft models rarely exhibit comparable efficacy in patients with the anatomically/pathologically equivalent tumor [5-7]. Indeed, it has been highlighted that the failure of drugs during clinical trials is linked to a lack of testing in clinically relevant preclinical models [8]. The advent of “precision medicine” (PMed), utilizing novel molecular techniques to design patient tumor-specific therapeutic regimens, requires preclinical cancer models that closely reflect the originating human disease to allow evaluation and rapid translation of these molecular based approaches to therapy selection in the clinic [9].

While there have been a growing number of studies reporting comparative chemotherapeutic responses between tumorgraft models and patients [10-13], there is limited data addressing genomic similarities between tumorgraft models and the originating patient tumors [14-16]. Previous studies have reported excellent concordance in expression profiles between patient tumors and tumorgraft models of similar histotypes [17,18]; however, the comparisons were not made in a pairwise fashion between each tumorgraft and the originating tumor from the donor patient.

Given the added cost and time required to develop primary patient tumorgrafts relative to the more classical cell line xenografts, a detailed cellular and molecular characterization of the tumorgraft models showing a high degree of equivalence to the originating patient’s tumor in the human host could provide the justification for the routine use of this model, even at the ectopic subcutaneous site, in translational studies and personalized medicine applications. Our goal was to compare the genomic profiles of a panel of diverse tumorgraft models to the patient tumor tissue from which the models were derived. To address inter-disease variability, the panel of tumorgraft models selected for this study contains a spectrum of neoplastic diseases, rather than a limited set of disease types [14-16,19]. Our long term objective is to develop this panel of mixed-type tumorgraft models to evaluate new treatment strategies based on the molecular characterization of an individual disease and the subsequent treatment with targeted therapy, independent of the tumor histology and anatomical location.

## Methods

### Human subjects

Ethical approval for this study was granted by the Institutional Review Boards at both the Van Andel Research Institute (VARI) and the collaborating clinical institutions: Spectrum Health Hospitals, Grand Rapids, MI; Saint Mary's Health Care, Grand Rapids, MI; Oncology Care Associates, St. Joseph, MI; Mary Crowley Cancer Research Centers, Dallas, TX. Written, informed consent was obtained from each patient or guardian prior to study enrollment. Eligibility for tumorgraft development was not limited by cancer type, cancer staging, or anatomical location. The tumor tissue used for tumorgraft development was deemed excess to that required for the patient’s diagnosis and standard of care and treatment.

### Tumor processing

The tumor tissue was subdivided into a portion for genomic analysis and a portion for implantation into immune-compromised mice. Tissue for genomic analysis was snap frozen (30–60 s) in chilled isopentane (dry ice) and stored at −80° C. Tumor tissue for implantation into mice was maintained in 6 ml transport media, [RPMI 1640 media (Invitrogen, Carlsbad, CA), 1% penicillin/streptomycin (Invitrogen), and 50 units of heparin (Sigma, St. Louis, MO)/ml media] at 4 °C until implantation. Samples from distant sites were shipped on wet ice and were implanted within 24 hours of surgical resection.

### Tumorgraft development

All animal studies were approved by the VARI Institutional Animal Care and Use Committee (IACUC) and used 6–8 week old athymic *nu/nu* mice from the VARI breeding colony. Food and water was available ad libitum for the duration of the studies. Mice for each tumorgraft model were gender matched to the donor patient. Body weights of the mice were recorded weekly during tumorgraft development. Tumorgraft volumes (½ x length x depth x height) were measured 1x/week when volumes ≤50 mm^3^ and 3x/week at tumor volume >50 mm^3^. Mice were euthanized and subcutaneous tumorgrafts harvested following IACUC guidelines.

Upon receipt, the tumor tissue for implantation was placed into a sterile dish containing sterile phosphate buffered saline (Invitrogen) and carefully teased into ≤3 millimeters (longest axis) tumor fragments. Dependent on tumor tissue availability, tumor fragments were implanted in a maximum of five mice (1^st^ generation). Following administration of general anaesthesia (isoflurane), the right flank was cleaned with 70% ethyl alcohol, a small incision made, and a subcutaneous pocket created by blunt dissection. The tumor fragment was inserted into the pocket and the incision closed using a surgical staple. Immediately following surgery, the mouse received a single dose of the analgesic Ketoprofen (5 mg/kg body weight). Mice were monitored for health and tumor growth for the duration of the study. A tumorgraft model that failed to develop within 6 months in the 1^st^ generation mice was discontinued and the mice euthanized.

When a 1^st^ generation tumorgraft reached a volume of ≥1500 mm^3^ the mouse was euthanized and the tumorgraft was aseptically harvested. The tumorgraft was subsequently divided into pieces for four applications: 1) 5 pieces (≤3 millimeters (longest axis) were directly transplanted into 2^nd^ generation mice; 2) 18 pieces (≤3 millimeters (longest axis) were cryopreserved (RPMI 1640 media (Invitrogen), 10% fetal bovine serum (Invitrogen), 10% dimethyl sulfoxide (Sigma, St. Louis, MO), and 50 units heparin (Sigma)/ml RPMI 1640 media), using a “Mr. Frosty” Freezing Container (Thermo Scientific, Waltham, MA) at −1°/min cooling rate, in a −80 °C freezer, to allow for subsequent reestablishment of the tumorgraft model; 3) a single piece (3 mm x 5 mm x 5 mm) was snap frozen for future genomic/proteomic analysis; and 4) remaining tissue was formalin fixed.

The 2^nd^ generation mice were monitored for health and tumorgraft growth characteristics as with the 1^st^ generation mice. Once a total of ~50 tumorgraft fragments, from either 1^st^ or 2^nd^ generation mice, had been cryopreserved, all remaining mice were euthanized and any tumorgrafts harvested (snap frozen and formalin fixed) and stored for subsequent analyses. Two osteosarcoma models were grown out to a 4^th^ generation and used to examine longitudinal changes such as genetic drift and mutation status.

For a tumorgraft model to be considered fully established the following three criteria needed to be met: 1) 1^st^ generation growth, 2) 2^nd^ generation tumor growth following direct (fresh) transplant of a tumor fragment from a 1^st^ generation mouse, and 3) re-growth of the cryopreserved tumor fragment. To test cryo-viability, three 1^st^ generation tumor fragments, frozen in liquid nitrogen for a minimum of four weeks, were thawed rapidly, rinsed in sterile phosphate buffered saline (Invitrogen) containing 1% penicillin/streptomycin (Invitrogen), and implanted into immune-compromised mice, as described above. In the event that the cryopreserved tissue failed to develop into a tumorgraft within 6 months, the model was deemed unsuccessful and the mice euthanized. While the preservation of the histopathological features of the primary patient tumor in the resulting tumorgraft models was assessed, it was not a requirement for a successful model development.

### Data storage and management

For this study, all clinical, preclinical, histological, and genomic data was stored and analyzed using the XenoBase-BioIntegration Suite (XB-BIS) (http://xbtransmed.com/). Data analysis is accomplished by integrating molecular, preclinical and clinical data, facilitating integrated analysis within this single platform as described in the pertinent sections below.

### Histological analysis of tumorgrafts and patient tumors

Histopathological evaluation was performed on five micron hematoxylin and eosin (H&E) stained sections of formalin-fixed paraffin-embedded (FFPE) pieces of patient tumors and of subsequent 1^st^ and 2^nd^ generation tumorgrafts. Tissue sections were reviewed by two independent pathologists (BJN & RS) to compare the pathological features of the tumorgrafts and compared directly with those of the corresponding patient tumor.

### Mutational analysis using the sequenom OncoCarta assay

For mutation detection, the OncoCarta mutation panel was used according to manufacturer’s protocol (Sequenom, San Diego, CA). The OncoCarta™ Panel v1.0 (Sequenom, San Diego, CA) has the capacity to detect 238 mutations in 19 oncogenes.

Ten representative pairs of 1^st^ generation tumorgrafts and originating patient tumors and 1^st^ - 4^th^ generation series of an osteosarcoma tumorgraft model were screened using the MassARRAY system and OncoCarta Panel v1.0 (Sequenom, San Diego, CA) [20,21]. Tumor DNA was isolated from either snap frozen of formalin-fixed paraffin embedded tissue using an initial Proteinase K digestion followed by salt ethanol precipitation. The DNA was further purified using the QIAamp DNA micro Kit (Qiagen, Valencia, CA). Only samples in which the H&E sections, from above and below the processed tissue, contained ≥50% of viable malignant cell content (assessed by nuclei vs. surface area) were submitted for analysis of oncogenic mutations.

The spectra from the MassARRAY Compact Analyzer were loaded into MassARRAY Typer Analyzer software 4.0.4.20 (Sequenom). The analysis software automates the identification of mutants by comparing ratios of the wild type peak to that of all suspected mutants and adjusting these peaks for adducts it detects in the spectrum [20,21]. All mutations detected by this automated process were visually reviewed to identify "real" mutant peaks and to remove all artifact peaks due to salt peaks or other background peaks.

### Gene expression analysis

Transcriptome profiling of the genomes of 24 representative 1^st^ generation tumorgraft/patient tumor pairs, encompassing gastrointestinal, musculoskeletal, skin, head and neck, and genitourinary cancers, and genomes of two musculoskeletal models across four tumorgraft generations was performed using the Human U133 Plus 2.0 GeneChip® (Affymetrix, Inc, Santa Clara, CA). The resulting gene expression data were stored and analyzed in XB-BIS. RNA was isolated from snap-frozen patient tumors and tumorgraft tissue that was assessed for viable malignant cell content, as described above.

RNA was isolated by chloroform extraction, precipitation in isopropyl alcohol, a 70% ethanol wash and suspension in nuclease free water. A final RNA purification was performed using RNeasy Mini-/Micro-Kit^TM^ (Qiagen) with an on-column DNase 1 digestion. RNA quantity and purity was determined using a NanoDrop Spectrophotometer (NanoDrop, Wilmington, DE); RNA quality was determined with an Agilent 2100 Bioanalyzer (Agilent, Foster City, CA).

Labeled one round cDNA was generated either by the Affymetrix method: Gene-Chip One-Cycle Target Labeling and Control Reagent (Affymetrix) or by the NuGEN method: WT-Ovation Pico RNA Amplification system and FL-Ovation cDNA Biotin Module (NuGEN Technologies, Inc., San Carlos, CA). The resulting amplification products were quantified and hybridized to GeneChip® Human Genome U133 Plus 2.0 Arrays (Affymetrix, Inc). Hybridization, washing, staining and scanning of the arrays were performed according to manufacturer’s instructions (Affymetrix, Inc.). The resulting .cel files were loaded into the Affymetrix Expression Console Version 1.1, batch processed using the Affymetrix MAS5 algorithm with a target value of 500. The resulting pivot table of normalized expression intensities was then exported into XB-BIS for subsequent analysis.

### Comparative analysis of genomes of tumorgrafts and matched patient tumors

Genomic similarity between the tumorgraft and its matched patient tumor were assessed by determination of Pearson correlation coefficients and by unsupervised cluster analysis. For determination of Pearson correlation coefficients, gene expression data was filtered to remove those Affymetrix probes deemed absent and/or with normalized intensities less than 100 in ≥50% of the sample set of 24 matched tumor/tumorgraft pairs (n = 25,806 probe sets); the gene expression data was not censored for Affymetrix x_at probes.

For unsupervised cluster analysis, the selection of the significant variance probes was made to emphasize the differences in the matched tumor pairs, which is diminished by large numbers of low intensity, non-significant probes that are similar both between pairs and across the tumor panel. The probe sets were filtered to include only probe sets with an intensity ≥150, called present in ≥20% samples, and exhibited significant variance (coefficient of variance >1). Affymetrix x_at probes were excluded from the final data used for the cluster analysis.

Initial unsupervised clustering of the 24 tumorgraft/patient tumors, identified that the choice of RNA amplification methods, Affymetrix and NuGEN, was a significant confounding variable (data not shown). Subsequently, the 48 data sets were clustered independently, based on method of RNA amplification (Affymetrix, 28 genomes; NuGEN, 20 genomes). The resulting Affymetrix amplification probe set (n = 6,845) and NuGEN amplification probe set (n = 6,265) were loaded into the programming language R version 2.11.1. The distance matrix was created using Euclidean distance default parameters and complete linkage with the default parameters were utilized for the hclust function. The unrooted dendrogram was constructed using the phylo object and plotted with default parameters.

To determine whether the differential expression in the genomes between the tumorgrafts and the originating patient tumors was random or represent a gain or loss of specific pathways, a paired t-test (p < 0.05) was employed on the set of 25,806 probes, generated above, to identify those genes that were differentially expressed, 2-fold or greater, between tumorgrafts and their matched tumors. No multiple testing correction was applied because the sequential pathway enrichment analysis is expected to remove or diminish the effects of random significant gene selection. The resulting genes up-regulated (n = 17) or down-regulated (n = 395) in tumorgrafts, as compared to the originating patient tumors (Additional file 1: Table S1), were analyzed for canonical pathway enrichment and ontology enrichment using the MetaCore™ pathway analysis suite (GeneGo-Thomson Reuters, St. Joseph, MI) using p-values of hypergeometric distribution as enrichment and ranking method [22]. GeneGo’s data import methods automatically resolve duplicate gene mapping and unmapped Affymetrix probes. The attrition in the mapping of the Affymetrix probes in Additional file 1: Table S1 ranged from 30% to 35%. In addition, the gene lists were analyzed for enrichment in broadly defined protein functions, i.e. transcription factors, secreted proteins, kinases, receptors, proteases, etc. using MetaCore™’s Interactome workflow.

### Analysis of genomes of tumors that successfully formed tumorgrafts compared to tumors that failed to form tumorgrafts

To begin to understand why all donor tumors do not form tumorgrafts following transplant into immune-compromised mice, the genomes of 16 patient tumors that did form tumorgrafts were compared to 16 tumors that did not form tumorgrafts. These 32 genomes were selected since they were all amplified by the Affymetrix method, thus removing the confounding effect of the RNA amplification method.

For the analysis of the tumorgraft-forming tumor genomes against the non-tumorgraft forming tumor genomes, a student’s two-tailed t-test (p < 0.05) was employed to identify genes that were differentially expressed, 2-fold or greater, between the two classes. No multiple testing correction was applied because the sequential pathway enrichment analysis is expected to remove or diminish the effects of random significant gene selection. The resulting list of up-regulated (n = 491) and down-regulated (n = 691) genes in the tumorgraft-forming tumors, compared to the non-tumorgraft forming tumors (Additional file 2: Table S2), were submitted to MetaCore™ for enrichment analysis and interaction analysis, as described above. The attrition in the mapping of the Affymetrix probes in Additional file 2: Table S2 ranged from 28% to 36%.

## Results

Forty-nine tumorgraft models, representing 18 distinct cancer pathologies have been developed by direct implantation of patient tumor tissue into immune-compromised nude mice giving the overall take rate of 27% (Table 1). These 49 models were distributed across eight different cancer location categories, based on the NCI Cancer by Body Location/System (Table 2). Tumorgraft models of the gastrointestinal tract and skin account for ~50% of successfully established tumorgraft models. There were an additional three models that formed 1^st^ generation tumorgrafts but that failed to re-establish tumorgrafts following cryopreservation of 1^st^ generation tumorgraft fragments, giving a 94% success rate in tumorgraft model re-establishment following cryopreservation. No association was identified between tumorgraft development and the time between surgical resection and implantation (p = 0.86 by simple t-test on representative sample set, data not shown).

**Table 1:** Summary of Tumorgraft Development

**Location**	**Tumor type**	**# Models**	**Success**	**Failure**	**Success (%)**
**Breast**	Breast Cancer	55	3	52	5
	**Total**	**55**	**3**	**52**	**5**
**Endocrine**	Neuroendocrine Tumor	3	1	2	33
	Papillary Thyroid Carcinoma	2	0	2	0
	**Total**	**5**	**1**	**4**	**20**
**Gastrointestinal**	Cecal Adenocarcinoma	1	1	0	100
	Cholangiocarcinoma	1	0	1	0
	Colorectal Cancer	17	8	9	47
	Esophageal Adenocarcinoma	5	2	3	40
	Gallbladder Carcinoma	1	0	1	0
	Gastric Carcinoma	3	1	2	33
	Hepatoblastoma	1	0	1	0
	Pancreatic Cancer	6	3	3	50
	Small Bowel Cancer	2	0	2	0
	**Total**	**37**	**15**	**22**	**41**
**Genitourinary**	Bladder Carcinoma	1	0	1	0
	Clear Cell Sarcoma	1	0	1	0
	Prostate Adenocarcinoma	1	0	1	0
	Renal Cell Carcinoma	4	0	4	0
	Urachal Adenocarcinoma	1	1	0	100
	Wilms Tumor	1	1	0	100
	**Total**	**9**	**2**	**7**	**22**
**Gynecologic**	Ovarian Carcinoma	2	0	2	0
	**Total**	**2**	**0**	**2**	**0**
**Head and Neck**	Head and Neck Cancer	2	2	0	100
	Nasopharyngeal Cancer	1	0	1	0
	Squamous Cell Carcinoma of the mouth	1	0	1	0
	**Total**	**4**	**2**	**2**	**50**
**Leukemia**	Acute Lymphoblastic Leukemia	1	0	1	0
	**Total**	**1**	**0**	**1**	**0**
**Lung**	Squamous Cell Carcinoma of the lung	5	0	5	0
	Lung Adenocarcinoma	4	1	3	25
	Small Cell Lung Cancer	1	0	1	0
	NSCLC	13	5	8	38
	**Total**	**23**	**6**	**17**	**26**
**Lymphoma**	Burkitts Lymphoma	1	0	1	0
	Follicular Lymphoma	3	0	3	0
	Hodgkins Lymphoma	1	0	1	0
	Lymphoangioma	1	0	1	0
	Lymphoma	6	0	6	0
	**Total**	**12**	**0**	**12**	**0**
**Musculoskeletal**	Desmoid Tumor	1	0	1	0
	Ewings Sarcoma	3	2	1	67
	Hemangiopericytoma	1	0	1	0
	Leiomysarcoma	1	0	1	0
	Osteosarcoma	3	3	0	100
	Rhabdomyosarcoma	3	1	2	33
	Synovial Sarcoma	1	1	0	100
	**Total**	**13**	**7**	**6**	**54**
**Neurologic**	Neuroblastoma	1	0	1	0
	**Total**	**1**	**0**	**1**	**0**
**Skin**	Melanoma	18	11	7	61
	Merkel Cell Carcinoma	2	2	0	100
	**Total**	**20**	**13**	**7**	**65**
**Final Total**		**182**	**49**	**133**	**27**

**Table 2:** Distribution of Tumorgraft Models across Cancer Location Categories and Staging

**Location**	**Tumor type**	**# Models**	**Staging**				
			**I**	**II**	**III**	**IV**	**ND**
**Breast**	Breast Cancer	3				2	1
**Endocrine**	Neuroendocrine Tumor	1		1			
**Gastrointestinal**	Cecal Adenocarcinoma	1	1				
	Colorectal Cancer	9	2			7	
	Esophageal Adenocarcinoma	2				2	
	Gastric Carcinoma	1				1	
	Pancreatic Cancer	3				3	
**Genitourinary**	Wilms Tumor	1					1
	Urachal Adenocarcinoma	1					1
**Head and Neck**	Head and Neck Cancer	2			2		
**Lung**	Lung Carcinoma	1					1
	NSCLC	5	1		2	2	
**Musculoskeletal**	Ewings Sarcoma	2					2
	Osteosarcoma	3			3		
	Rhabdomyosarcoma	1				1	
	Synovial Sarcoma	1					1
**Skin**	Melanoma	11			4	6	1
	Merkel Cell Carcinoma	2	1			1	
**Total**		**49**					

The take rate (the ability of donor tumor to successfully be propagated in mice) was closely related to tumor stage at time of resection. Forty-three percent (35/82) of tumors with a pathological staging of Stage III and Stage IV tumors were successfully propagated in mice, accounting for 73% of fully developed models (Table 2). Only 10% (6/60) of the donor tumors from Stage I or II cancers successfully propagated formed tumorgrafts in mice, accounting for only 12% of the fully developed models. Pathological staging information for the remaining 22% of the tumor tissues implanted into mice (40/182) which formed tumorgrafts was not obtained. A contingency table analysis with stage III and IV as one condition and stage I and II the second condition supported the hypothesis with a p-value = 1.2 E-5 and an odds ratio of 6.7 for this data set (data not shown).

Tumorgraft development was also found to be related to cancer type. Of the human tumor samples used to derive the panel of 49 tumorgraft models, a success rate of >40% was observed for skin (65%), musculoskeletal (54%), head and neck cancers (50%), and gastrointestinal cancers (41%) (Table 1). Lung (26%), genitourinary (22%), and endocrine (20%) cancers exhibited lower take rates for tumorgraft formation. Cancers of the breast (5%), predominantly early stage (data not shown), and the lymphatic system (0%) proved the most challenging to form tumorgrafts using the athymic nude mouse strain and methodologies adopted here. No tumorgrafts were established from tumors of gynecologic, leukemic, or neurologic origins; however, these finding are not necessarily representative of the true take rate for these tumor types as ≤2 attempts for each of these cancer types were attempted.

**Figure 1:**
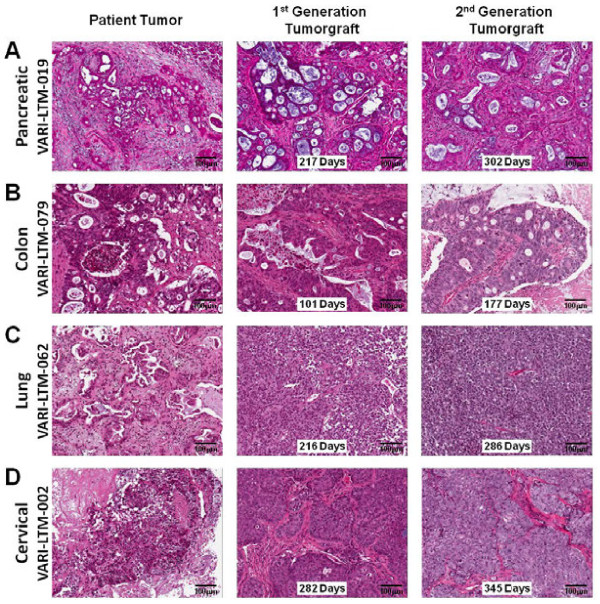
**Histological resemblance between patient tumor and resulting tumorgraft following sequential grafting into immunocompromised mice.** Representative sections stained with hematoxylin and eosin of indicated tumor samples (Scale bars indicate 100 μm). **A**) Adenocarcinoma of pancreas with desmoplastic stromal reaction in original patient tumor. 1^st^ generation tumorgraft (total time *in vivo*, 217 days) with preservation of atypical glandular structures with inspissated mucinous material surrounded by fibrous stroma. 2^nd^ generation tumorgraft (303 days) with similar histology. **B**) Adenocarcinoma of colon with numerous atypical glands containing mucinous material and necrotic debris with surrounding fibrovascular connective tissue. 1^st^ generation tumorgraft after 101 days reveals dilated atypical ducts with mucin and necrotic debris in lumens comprised of multiple columnar layers of atypical epithelium surrounded by fibrovascular stroma. 2^nd^ generation graft after 177 days with similar histology. **C**) Lung carcinoma with prominent fibrovascular connective tissue in original patient neoplasm that becomes progressively overgrown by malignant cells in 1^st^ (216 days) and 2^nd^ (286 days) generation tumor grafts. **D**) Cervical carcinoma with fibromucinous stroma in original patient neoplasm, with expansion of large islands of malignant cells surrounded by fibrovascular stroma in 1^st^ (282 days) and 2^nd^ (345 days) generation tumorgrafts.

Hematoxylin and eosin stained sections from matching patient tumors and 1^st^ and 2^nd^ generation tumorgrafts from pancreatic cancer, breast cancer, lung cancer and cervical cancer models were examined for histological similarities. In all 4 different tumorgraft models, the initial patient tumors were characterized by histological features consistent with the anatomical site of origin (Figure 1 A-D). The 1^st^ generation tumor grafts resembled the original patient tumor, including in most instances retention of peritumoral stroma which became progressively replaced in the 2^nd^ generation tumorgrafts by malignant cell overgrowth. In addition, the relative state of differentiation of the respective cancer type was retained following engraftment, including production of mucin and retention of atypical glandular structures for the adenocarcinomas, even after 302 and 177 days for the pancreatic and breast cancers, respectively. In the lung cancer and cervical cancer models, the 1^st^ and 2^nd^ generation tumors revealed viable malignant appearing cells. The 2^nd^ generation tumorgrafts retained essential diagnostic features at 286 and 345 days, respectively, for the lung and cervical cancer models.

Of the 20 samples (10 patient samples, 10 1^st^ generation tumorgraft samples) submitted for oncogene mutation analysis, only 15 mutations were identified. Four tumorgraft models each had a single point mutation that was also observed in the originating tumor tissue. Models VARI-LTM-044 colorectal cancer and VARI-LTM-034 pancreatic cancer both had a single KRAS mutation; melanoma models VARI-LTM-086 and VARI-LTM-027 had BRAF and NRAS mutations, respectively (Table 3). Model VARI-LTM-041 colorectal cancer contained three mutations (KRAS and two PIK3CA mutations) that were also present in the matching tumor. The colorectal cancer model VARI-LTM-026, did not exhibit mutations in the patient tumor, but showed a single KRAS mutation in the tumorgraft. In the osteosarcoma model VARI-LTM-021, no mutations were identified in the patient tumor with any genetic drift identified in the 1^st^ - 4^th^ generation tumorgrafts (data not shown).

**Table 3:** Oncogene mutations detected in tumorgrafts and originating patient tumors

**Model**	**Tumor Type**	**Gene**	**Mutation**	**Primary Tumor Mutation Status**	**Tumorgraft Mutation Status**
VARI-LTM-041	Colorectal Cancer	KRAS	G12V	TRUE	TRUE
VARI-LTM-041	Colorectal Cancer	PIK3CA	R88Q	TRUE	TRUE
VARI-LTM-041	Colorectal Cancer	PIK3CA	E545K	TRUE	TRUE
VARI-LTM-044	Cecal Adenocarcinoma	KRAS	G13D	TRUE	TRUE
VARI-LTM-026	Colorectal Cancer	KRAS	G12D	FALSE	TRUE
VARI-LTM-086	Melanoma	BRAF	V600E	TRUE	TRUE
VARI-LTM-027	Melanoma	NRAS	Q61R	TRUE	TRUE
VARI-LTM-034	Pancreatic Cancer	KRAS	Q61H	TRUE	TRUE

Paired analysis of the 24 tumorgraft/tumor pairs exhibited a high degree of similarity, with Pearson correlation coefficients ranging from r = 0.75 to 0.99 with a median coefficient of 0.93 (Table 4). There were only four pairs, three gastrointestinal and one lung, that had Pearson values r < 0.90; the NSCLC model VARI-LTM-023 had the Pearson correlation coefficient of r = 0.75. For this model, the originating patient tumor RNA had a RIN (RNA Integrity Number) of 4.9 implying degraded RNA, which was likely to be the primary factor driving the low correlation and would typically be excluded from the molecular analysis.

**Table 4:** Pearson correlation coefficient of each individual tumorgraft and originating patient tumor based on Affymetrix gene expression data

**Cancer Location Category**	**Tumor Type**	**Subject ID**	**Pearson correlation (R)**
**Gastrointestinal**	Cecal Adenocarcinoma	VARI-LTM-044	0.87
	Colorectal Cancer	VARI-LTM-026	0.86
	Colorectal Cancer	VARI-LTM-034	0.97
	Colorectal Cancer	VARI-LTM-041	0.99
	Gastric Carcinoma	VARI-LTM-024	0.86
	Pancreatic Cancer	VARI-LTM-035	0.92
**Genitourinary**	Wilms Tumor	VARI-LTM-022	0.98
**Head and Neck**	Head and Neck Cancer	VARI-LTM-042	0.94
	Head and Neck Cancer	VARI-LTM-045	0.90
**Lung**	Lung Cancer	VARI-LTM-029	0.95
	NSCLC	VARI-LTM-023	0.75
	NSCLC	VARI-LTM-046	0.93
	NSCLC	VARI-LTM-038	0.93
**Musculoskeletal**	Ewings Sarcoma	VARI-LTM-028	0.97
	Osteosarcoma	VARI-LTM-020	0.93
	Osteosarcoma	VARI-LTM-021	0.95
	Osteosarcoma	VARI-LTM-031	0.93
	Rhabdomyosarcoma	VARI-LTM-058	0.94
	Synovial Sarcoma	VARI-LTM-019	0.91
**Skin**	Melanoma	VARI-LTM-025	0.96
	Melanoma	VARI-LTM-027	0.92
	Melanoma	VARI-LTM-043	0.97
	Merkel Cell Carcinoma	VARI-LTM-033	0.97
	Merkel Cell Carcinoma	VARI-LTM-039	0.93

**Figure 2:**
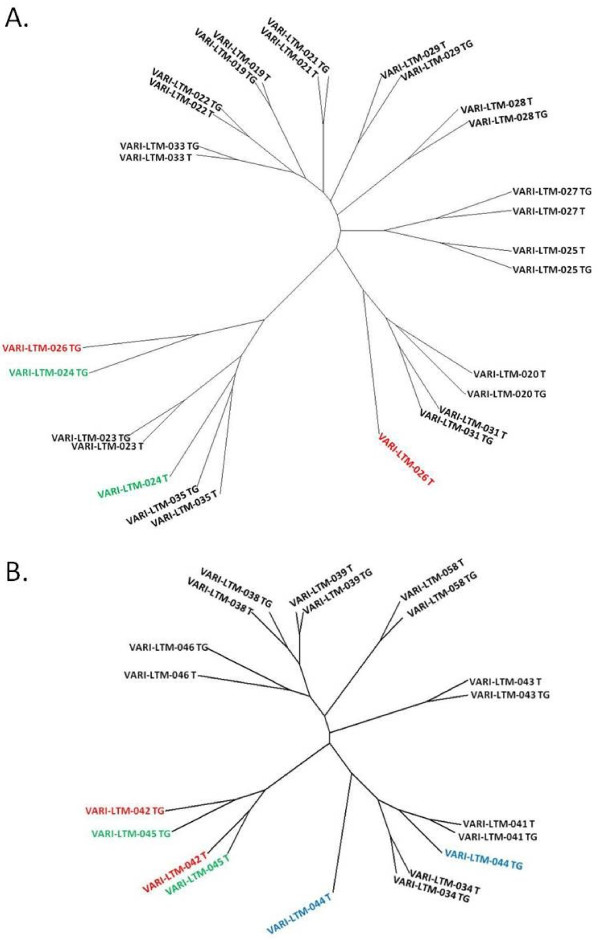
**Unsupervised cluster analysis of genomes of tumorgrafts and originating patient tumors.** Unsupervised clustering was performed on genomes amplified by **A**) Affymetrix method, 28 genomes total; and **B**) NuGEN method, 20 genomes total. Clustering was performed using programming language R version 2.11.1 on normalized gene expression data that was pre-processed using the Affymetrix MAS5 algorithm to an average intensity target of 500. Other than a few highlighted outliers discussed in the text, patient tumors and their derived tumorgrafts are closely related at the gene expression level.

Of the 28 genomes amplified by the Affymetrix method, 12 of the 14 tumorgraft/donor tumor pairs co-clustered in an unsupervised analysis (Figure 2A). Of the 20 genomes amplified by the NuGEN method, 7 of 10 tumorgraft/ tumor pairs coclustered (Figure 2B). 4 of the 5 tumorgraft/tumor pairs that did not co-cluster in an unsupervised fashion exhibited Pearson correlation coefficients r ≤ 0.90 (Table 4). The tumorgraft and originating patient tumor from the NSCLC model VARI-LTM-023 co-clustered despite a low Pearson correlation coefficient of r = 0.75, suggesting significant variance in respect to other tumors in the cohort.

The genomic profiles of two representative osteosarcoma models VARI-LTM-020 and VARI-LTM-021 were observed over 4 generations to assess the potential for genomic drift over time in vivo. The Pearson correlation coefficients were very high, ranging from r = 0.98-0.99 between all 4 generations in both models (data not shown). The largest decrease in Pearson correlation coefficient occurred between the human tumor to 1^st^ generation mouse tumorgraft (VARI-LTM-020, r = 0.91; VARI-LTM-021, r = 0.94). Genomic fidelity was then maintained in subsequent mouse to mouse generation comparisons. This stability in the genomes across mouse tumorgraft generations, along with the absence of additional somatic oncogene mutations suggests the maintenance of genomic stability during serial *in vivo* passages and time *in vivo* for at least up to 186 days (VARI-LTM-021).

**Figure 3:**
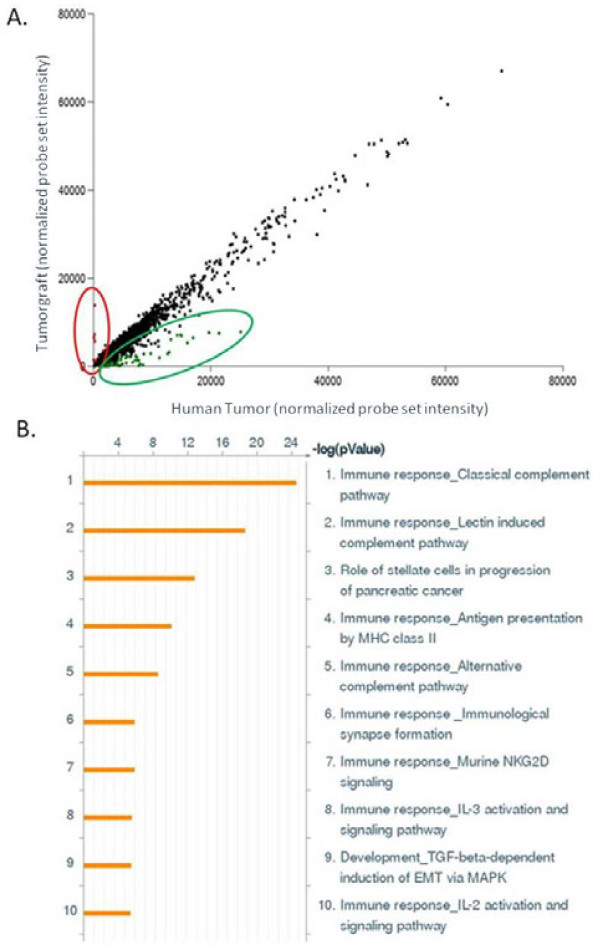
**Enrichment of canonical pathway maps in genes downregulated in tumorgrafts compared to their donor tumors.A**) Scatter plot of expression of gene probes (25,806) graphically depicting the up-regulated (red circle) and down-regulated (green circle) 2-fold or greater (t-test, p ≤ 0.05) in tumorgrafts relative to the originating patient tumors. **B**) Ontology enrichment analysis was performed using the MetaCore™ pathway analysis suite (GeneGo-Thomson Reuters, St. Joseph, MI). Eight of the top ten canonical pathways enriched in the genes down-regulated in the tumorgrafts, relative to the originating patient tumors, were immune-related, consistent with immunodeficient status of the murine host.

Paired analysis of all 24 tumorgrafts to their matching tumors identified 25,806 gene probes that were present in at least 50% of the 48 samples. 395 genes were decreased and 17 genes increased two-fold or greater in the tumorgrafts (Additional file 1: Table S1) compared to the patient tumors (Figure 3A, green and red circles respectively).

Of the 395 genes down-regulated in the murine tumorgrafts, there was a significant enrichment in pathways related to immune response (Figure 3B). Enrichment scores were extremely high, with the top-scored classical complement pathway showing a –log p-value of 25. Of the 17 genes up-regulated in the tumorgrafts, there were only 1–2 genes per map, suggesting no enrichment for any canonical pathway (data not shown). An independent GO biological pathway enrichment analysis was conducted using the down regulated probe list and 14 of the most significantly enriched top 100 pathways p < 4.0E-9 were associated with immune response [23]. Classification of the down-regulated genes with regards to enrichment in protein function, identified a borderline significance (p = 0.05) in receptors and ligands in the tumorgrafts compared to the patient tumors. For the up-regulated genes, the only significant enrichment in protein function was for proteases, accounting for only two of the 17 genes.

**Figure 4:**
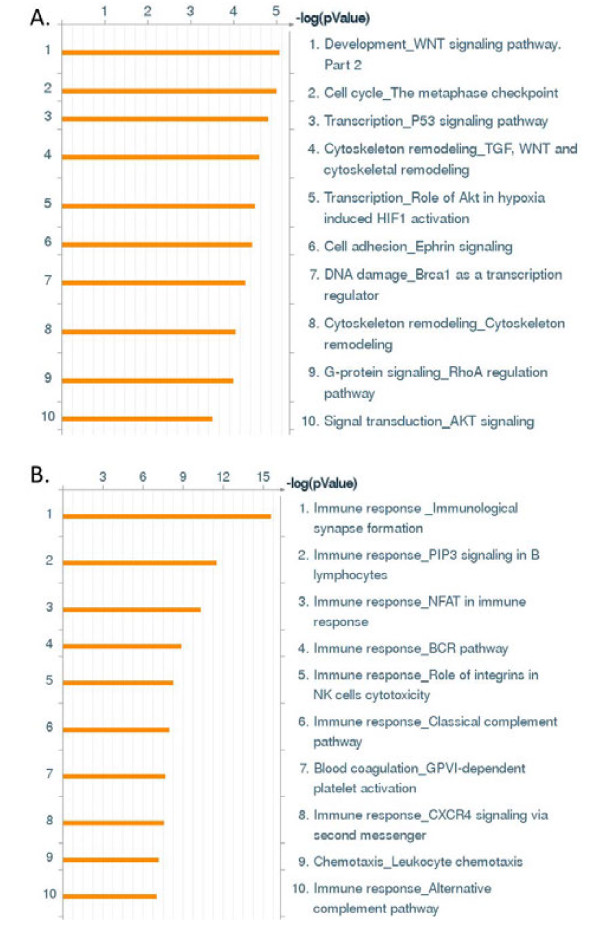
**Enrichment of canonical pathway maps in genes up and downregulated in tumorgraft forming tumors compared to non-tumorgraft forming tumors.** Ontology enrichment analysis was performed using the MetaCore™ pathway analysis suite (GeneGo-Thomson Reuters, St. Joseph, MI). **A**) The top ten canonical pathway maps enriched in the genes up-regulated (n = 491) in the tumors that formed tumorgrafts included cell signalling and cell cycle-related pathways. **B**) Eight of top ten canonical pathway maps enriched in the genes down-regulated (n = 691) in the tumorgrafts that formed tumorgrafts were immune-related pathways.

**Figure 5:**
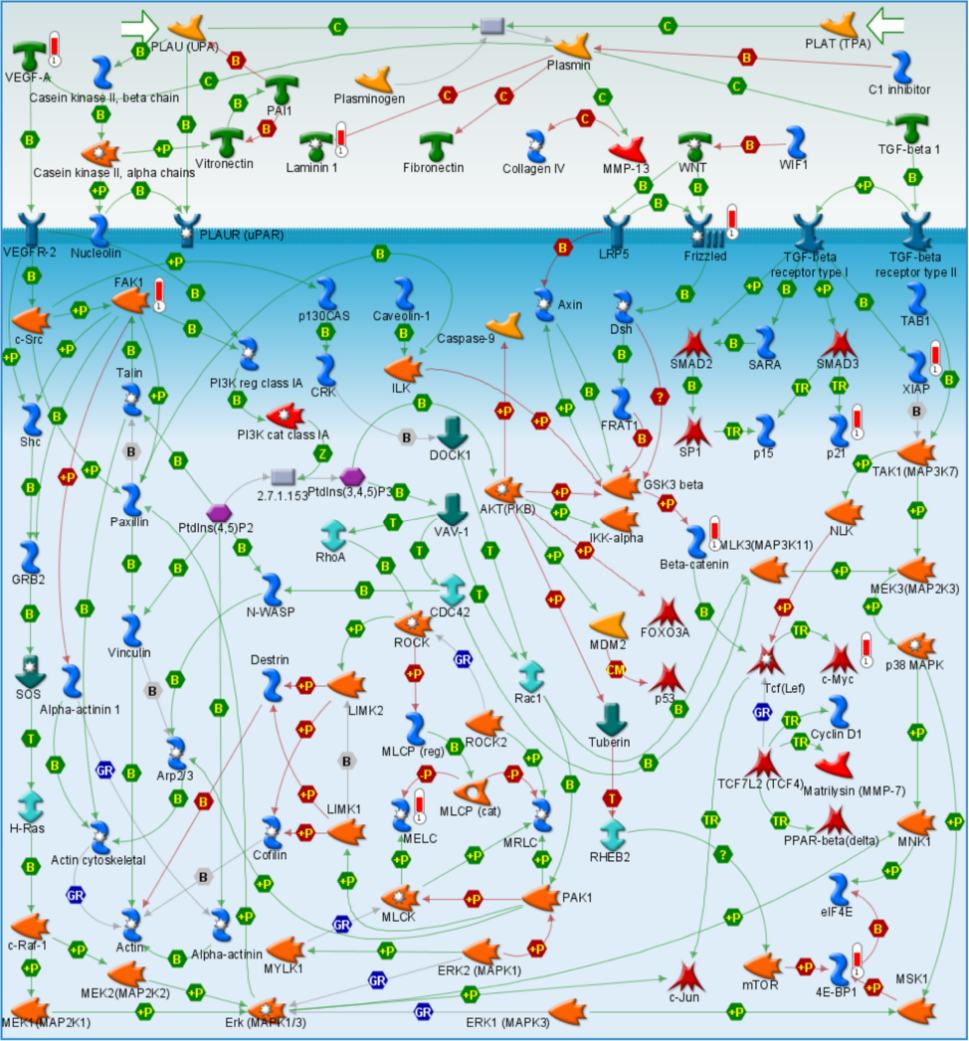
**WNT signalling pathway map enriched with genes up-regulated in tumorgraft-forming tumors.** The MetaCore™ pathway analysis suite (GeneGo-Thomson Reuters, St. Joseph, MI) identified a pathway map for WNT signalling. VEGF-A, Frizzled, β-catenin, FAK-1, c-Myc, Laminin 1, XIAP, p21, MELP, and 4E-BP1 present in this pathway were all up-regulated in tumorgraft forming tumors.

**Figure 6:**
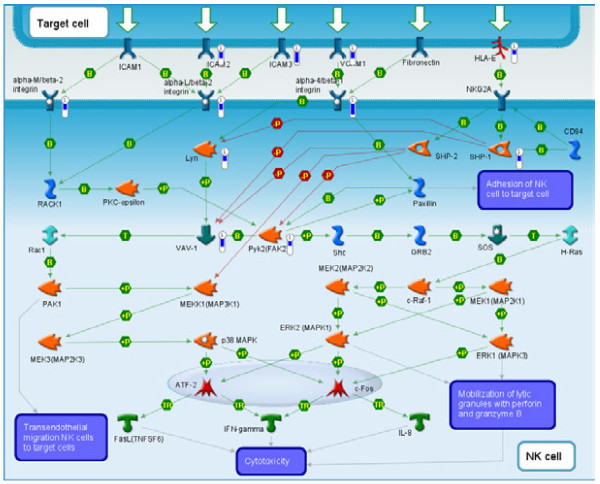
**Immune response pathway map enriched with genes down-regulated in the tumorgraft forming patient tumors.** The MetaCore™ pathway analysis suite (GeneGo-Thomson Reuters, St. Joseph, MI) identified a pathway map for an immune response involving integrins in NK cell cytotoxicity that was enriched for several ligands down-regulated in tumors that did not form tumorgrafts (Target cell denotes tumor cells).

Comparative analysis of the patient tumors that formed tumorgrafts to patient tumors that did not form tumorgrafts identified 491 genes that were up-regulated and 691 genes down-regulated 2-fold or greater in those tumors that formed tumorgrafts compared to non-tumorgraft forming tumors (Additional file 2: Table S2). The up-regulated genes in the tumors that formed tumorgrafts were enriched for cell cycle, cell signaling pathways and cytoskeleton remodeling (Figure 4A). One key canonical pathway map, enriched with up-regulated genes that were elevated in those tumors that formed tumorgrafts, is for WNT signaling (Figure 5); genes up-regulated in this pathway map included VEGF-A, Frizzled, β-Catenin, c-Myc, and FAK-1. The down-regulated genes in the tumorgraft-forming tumors were enriched for immune response pathways (8/10 pathways, Figure 4B). One key pathway map enriched with down-regulated genes is that of the role of integrins in NK cell cytotoxicity (Figure 6); note that in the context of this study, the upper Target Cell in this pathway corresponds to the relevant tumorgraft-forming cells.

## Discussion

The goal of this study was to create a panel of tumorgraft models, developed directly from patient tumor tissue from a wide range of heterogeneous tumor types of various stages. Prior investigations have focused on specific cancers, e.g. Secondary liver cancer [15] lung [24], pancreatic cancer [16], pediatric osteosarcomas [14], or pediatric rhabdomyosarcoma [25], rather than a spectrum of malignancies. To examine the suitability of the panel of tumorgraft models generated in our lab as molecularly-relevant preclinical models, we directly compared a number of phenotypic and genotypic features of the tumorgrafts to the originating patient tumors from which they were derived.

We have fully developed 49 models, spanning 18 different cancer types. A critical component of our development process is the ability of the model to re-establish following cryopreservation, alleviating the need for continual propagation, reported by some investigators [16]. Cryopreservation significantly reduces the cost and necessary resources, extending the life of the tumor model, and minimizing genetic drift that could occur following long-term continuous *in vivo* propagation.

Our overall tumor take rate is similar to previous reports [3,5]. A number of strategies have been tested to improve tumorgraft take rates, including the severe combined immunodeficient mouse strains CB17SC-M-F *scid*^*−/−*^[9,25,26] and CB17/Icr *scid*[12], suppression of the immune system of recipient CBA/CaJ mice by thymectomy, whole body irradiation, administration of 1-β-D-arabinofuranosylcytosine [14,25], and use of orthotopic models [27]. However, these strategies could increase the cost of model development with minimal improvement in tumorgraft development over the use of subcutaneous implantation into naive athymic *nu/nu* mice reported in this study and by other investigators [2,16].

It should be noted use of the athymic *nu/nu* mouse for use in the development of such tumorgraft does have limitations. The use of more immunodeficient mouse strains, e.g. NOD/*scid* or NOD/s*cid* gamma (NSG), are necessary for successful engraftment of certain tumor types e.g. leukemia [28,29], may better resemble the human microenvironment, and could provide better consistency in growth rates following implantation of human tumor tissue into mice. However, in the context of developing a panel of tumorgraft models across a wide range of tumor types, the athymic *nu/nu* mouse has proven a robust model.

The stage of the cancer at the time of acquisition has an impact on a successful tumorgraft development. Only 10% of Stage I and II tumors successfully formed tumorgrafts following implantation into mice; this is in contrast to the 43% take rate of tumor tissue from patients with Stage III or IV cancer at time of tissue acquisition which was successfully propagated in the murine host. Overall, ~75% of the 49 tumors that formed tumorgrafts were from patients that were diagnosed either Stage III or IV.

In this study, breast cancers had a take rate of only 6% following subcutaneous implantation into mice. Dobrolecki et al [27] reported success in developing breast cancer models by orthotopically implanting the breast cancer tissue into the mammary fat pads of mice. While more labor intensive than the subcutaneous model, an orthotopic approach should be considered if there is need for a tumorgraft model of a specific cancer that cannot be established using the subcutaneous model.

An important conclusion from this study is that these subcutaneous (predominantly ectopic with the exception of melanomas) tumorgraft models recapitulate the expression profiles of the original patient’s tumor within its natural site of site of metastatic spread. This provides some evidence that refutes the lack of relevance of the subcutaneous model for drug discovery and development, which may rather reflect the use of genetically drifted in vitro cell lines in the classical xenograft context. The primary value for any tumor model is to recapitulate the in-vivo malignant tissue state, and accurately predict the efficacy of therapeutic agents in a timely and cost-effective manner. In contrast with commonly used animal models in which established human cancer cell lines are injected subcutaneously and fail to accurately predict human clinical trial results [8], this human tumorgraft model system is more likely to be a successful surrogate preclinical model for several reasons.

First, tumor bearing tissue fragments are transplanted containing not only malignant cells, but also supporting stromal tissue in an anatomically correct hierarchy. Second, the transferred tumors include cells post engraftment retaining their essential cell to cell interactions and relative state of differentiation as assessed by their histological appearance. Third, because of the high success rate in establishing not only 1^st^, but also subsequent generation tumorgrafts, there is expansion of the number of tumorgrafts available for testing and the ability to verify retention of histological hallmarks in a serial fashion. Fourth, assessment of tumor growth is relatively straightforward given the subcutaneous location of the xenografts. Finally, since portions of tumors can be cryopreserved and successfully engrafted post thawing, testing of therapeutic agents, or combination therapies not originally considered, can be studied with this experimental and scalable *in vivo* model system.

In the era of molecularly targeted oncology agents, the effective translation of results in the mouse model to the clinic requires the genome of the tumorgraft model to be high comparable to the original tumor [30]. Significant changes in DNA fidelity or RNA expression levels significantly diminish the translational value of a model. Entire genome characterization of RNA expression rather, than a focused RT-PCR analysis of a select cohort of genes [16], allows for the assessment of transcriptome wide alterations and associated biological systems that occur during the growth of the transplanted human tumors.

The high Pearson correlations seen between the genomes of the 24 pairs of tumorgraft and originating patient tumors implies that the donor tumor genome is largely maintained in the tumorgraft model. Similarly, genotypic fidelity has also recently been reported in a panel of 25 human breast cancer tumorgrafts [27] and on a smaller scale in secondary liver cancers using quantitative PCR analysis of 21 genes related to oncogenesis and cell cycle [15]. The lower Pearson correlations observed in the gastrointestinal cancers could be an artifact of the tissue harvest and the high levels of digestive enzymes present in the patient tumors within the gastrointestinal tract. High levels of RNases present in pancreatic cancer are reported to complicate the extraction of high quality RNA [31]. This finding highlights the importance of an optimized, rapid tissue collection protocol, especially for genomic profiling. It should be noted that poor RNA quality, for genomic profiling does not necessarily impact the ability of the tissue to form a viable tumorgraft. The high degree of clustering by matched tumor/tumorgraft pairs (79%) supports the high degree of similarity of the genomes of the patient tumor and resulting mouse tumorgraft even when the opportunity for RNA degradation may occur.

Genomic stability is an important characteristic to consider prior to the long term use of a tumorgraft model. The high correlation in gene expression across four tumorgraft generations observed in this study and similar findings of others [3,16,27,30] and absence of drift in somatic mutations in known oncogenes, suggest that the genomes of tumorgraft models are stable. Histological integrity of patient-derived tumorgrafts has also been demonstrated for up to ten generations in immune-compromised mice [4] and up to 30 passages in mice without significant changes in growth and morphological characteristics [15]. One study reported tumorgraft models cultured *in vivo* for 10–12 generations before regenerating the models from earlier cryopreserved generations [3].

The role of somatic oncogene mutations in tumorigenesis, pathogenesis and disease progression can have a profound influence on therapy [32]; mutations in oncogenes RAS [33] and B-RAF [34] are commonly found in a variety of cancers. Therefore it is imperative that such mutations are maintained in the tumorgraft models. Although only a few oncogenic mutations were observed, those identified in the patient tumors were conserved in the matching tumorgraft, consistent with other reports [16] and consistent with known mutations in specific tumor types [20,21].

There are a number of possible explanations for the enrichment of canonical immune pathways within those genes down-regulated in the tumorgrafts compared to patient tumors. First, a loss of tumor cell immune pathways and proteins not necessary for growth and development when transplanted from the immunecompetent patient into the immunocompromised host. Second, there is an intentional tumor tissue response to transplantation to down regulate those genes responsible for its immunogenic signature presented to the vestigial immune defenses of the immunocompromised mouse. A third explanation, and most likely, is that the gene expression changes are due to a loss of circulating human immune cells which infiltrate and support tumor development, when the tumor was transplanted from the human into the mouse as earlier reported by Neale et al [17].

In those patient tumors that formed tumorgrafts, up-regulated genes show enrichment in pathways considered “usual suspects” in highly proliferative, late stage tumors; these canonical pathways include cell cycle, cytoskeletal remodeling, and those known drivers of tumorigenesis WNT and AKT signaling [35-37]. These same tumors were elevated for vascular endothelial growth factor (VEGF). This increased level of VEGF in the tumor could support the neovascularization of the tumor once implanted into the mouse; Nisolle et al [38] reported that the survival and growth of human endometrium transplanted into nude mice was correlated with a high VEGF content. Transgenic expression of VEGF into human islet cells followed by transplant into the livers increased the rate of tissue revascularization, survival and function [39]. A recent study demonstrated the pre-treatment of human ovarian tissue with VEGF and vitamin E prior to implantation into the back muscle of immunodeficient mice demonstrated enhanced growth, compared no pre-treatment of the ovarian tissue [40].

The downregulation of immunological pathways in the tumors that formed tumors would suggest that loss of immune pathways is a mark of late stage tumors and provides a selective advantage supporting the establishment of the tumors in a foreign host or location. This downregulation of immunological pathways could be a reflection of the loss of expression signature of patient immune infiltrating cells [17]. Of particular note is the downregulation of natural killer (NK) cell ligands, as identified as a pathway enriched for genes down-regulated in tumors that formed tumorgrafts compared to those tumors that failed to form tumorgrafts (Figure 4B). Although athymic *nu/nu* mice cannot generate mature T lymphocytes, they maintain the ability to mount a response to T-independent antigens [41] and do produce NK cells as a component of their vestigial immune system. The downregulation of the NK ligands may therefore promote human tumor development in immune compromised mice. While there is little evidence demonstrating the interaction of human integrins and murine NK receptors, Kanwar et al reports interspecies cross-talk via the demonstration that the ectopic expression of human intercellular cell adhesion molecule-1 (ICAM-1) in mouse EL-4 tumors inhibited *in vivo* tumor growth [42]. The mechanisms of NK cell recognition of self and immunosurveillance in both mouse and humans are complex and involve numerous receptors that recognize many cell surface ligands [43]. Experimental evidence clearly supports the view that murine NK cells can inhibit human tumor cell development and metastasis both *in vitro* and *in vivo*[44-50].

This study reports the development of a series of tumorgraft models, representing a broad spectrum of cancer types, derived from molecularly characterized fresh patient tumor tissue. The genomes of these tumorgraft models exhibit a high degree of correlation to their originating patient tumors which is maintained over multiple generations of *in vivo* passages. The loss of immune function pathways and gain of cell proliferation pathways suggests a logical selective pressure for certain tumors to form tumorgrafts, which could be prescreened in future studies to identify those most likely to develop into useful preclinical models for translational studies.

## Conclusions

The maintenance of the genome in the tumorgraft, from the human to murine host, supports the concept that tumorgraft models can provide an invaluable and robust tool in the evaluation of precision medicine methodologies and novel treatment strategies prior to use in clinical trials. An important point is that a subcutaneous primary patient tumorgraft models can recapitulate the expression profiles of the original “orthotopic” disease in the human and allow for reasonably high-throughput preclinical screens for drug efficacy. These models may therefore be useful in reducing the rate of attrition in drug development that has been in part been attributed to the use of the classical cell line xenografts.

## Competing interests

CPW has disclosed a potential conflict of interest in relation to his invention of the XB-BIS informatics system used to analyze some of the data presented in this manuscript. He is also a consultant for the company TransMed Systems who acquired licensing rights to XB-BIS. Some third parties have acquired some of the described tumorgraft models for commercial projects, but these remain available for academic (noncommercial) purposes.

## Authors’ contributions

DJM supervised the animal work, analyzed the data, interpreted the results, and wrote the manuscript. NRM interpreted the results and revised the manuscript. DMC, PJR, AI, and YN analyzed the data. DD and SBS carried out the animal work. EE, HF, SS, and REE carried out the laboratory experiments. JR, RS, and BN analyzed the histopathological images. CPW defined the research project, interpreted the results, and revised the manuscript. All authors read and approved the final manuscript.

## Supplementary Material

Additional file 1 **Table S1. The spreadsheet contains the results of the differential gene expression analysis comparing each patient’s tumor with the paired tumorgraft.** This file contains two spreadsheet tabs. The first tab displays the 17 gene probesets up-regulated in tumorgrafts (Group 2, column C) relative to the originating patient tumors (Group 1, column B), and the second tab displays the 395 gene probes down-regulated. The official gene name, mean normalized intensity in Groups 1 and 2, the associated fold-change in gene expression (Group 2/Group 1), the calculated pairwise t-test statistic, the corresponding p-value, the False Discovery Rate, and the Affymetrix probeset identifiers are shown. (XLSX 59 kb) Click here for file

Additional file 2 **Table S2. The spreadsheet contains the results of the differential gene expression analysis comparing the patient tumors that successfully developed tumorgrafts with those that did not.** This file contains two spreadsheet tabs. The first sheet displays the 491 gene probesets up regulated in the tumors that successfully formed tumorgrafts (Group 2, column C) relative to those that did not (Group 1, column B) and the second sheet displays the 691 gene probes down regulated. The official gene name, mean normalized intensity in Groups 1 and 2, the associated fold-change in gene expression (Group 2/Group 1), the calculated student’s t-test statistic, the corresponding p-value, the False Discovery Rate, and the Affymetrix probeset identifiers are shown. (XLSX 137 kb) Click here for file

## References

[B1] Cobb LM The behaviour of carcinoma of the large bowel in man following transplantation into immune-deprived mice Br J Cancer 1973 28 400 411 10.1038/bjc.1973.165 4357133 PMC2008916

[B2] Fiebig HH Schuchhardt C Henss H Fiedler L Lohr GW Comparison of tumor response in nude mice and in the patients Behring Inst Mitt 1984 74 343 352 6477362

[B3] Garber K From human to mouse and back: 'tumorgraft' models surge in popularity J Natl Cancer Inst 2009 101 6 8 19116380 10.1093/jnci/djn481

[B4] Houghton JA Taylor DM Maintenance of biological and biochemical characteristics of human colorectal tumours during serial passage in immune-deprived mice Br J Cancer 1978 37 199 212 10.1038/bjc.1978.28 629858 PMC2009589

[B5] Fiebig HH Widmer K-H Fiedler L WIttekind C Lohr GW Development and Characterization of 51 Human Tumor Models for Large Bowel, Stomach and Esophageal Cancers Dig Surg 1984 1 225 235 10.1159/000171659

[B6] Johnson JI Decker S Zaharevitz D Rubinstein LV Venditti JM Schepartz S Kalyandrug S Christian M Arbuck S Hollingshead M Sausville EA Relationships between drug activity in NCI preclinical in vitro and in vivo models and early clinical trials Br J Cancer 2001 84 1424 1431 10.1054/bjoc.2001.1796 11355958 PMC2363645

[B7] Voskoglou-Nomikos T Pater JL Seymour L Clinical predictive value of the in vitro cell line, human xenograft, and mouse allograft preclinical cancer models Clin Cancer Res 2003 9 4227 4239 14519650

[B8] Ellis LM Fidler IJ Finding the tumor copycat. Therapy fails, patients don't Nat Med 2010 16 974 975 10.1038/nm0910-974 20823880

[B9] Morton CL Houghton PJ Establishment of human tumor xenografts in immunodeficient mice Nat Protoc 2007 2 247 250 10.1038/nprot.2007.25 17406581

[B10] Boven E Winograd B Berger DP Dumont MP Braakhuis BJ Fodstad O Langdon S Fiebig HH Phase II preclinical drug screening in human tumor xenografts: a first European multicenter collaborative study Cancer Res 1992 52 5940 5947 1394220

[B11] Hidalgo M Bruckheimer E Rajeshkumar NV Garrido-Laguna I De Oliveira E Rubio-Viqueira B Strawn S Wick MJ Martell J Sidransky D A Pilot Clinical Study of Treatment Guided by Personalized Tumorgrafts in Patients with Advanced Cancer Mol Cancer Ther 2011 8 1311 1316 21673092 10.1158/1535-7163.MCT-11-0233 PMC4629061

[B12] Peterson JK Tucker C Favours E Cheshire PJ Creech J Billups CA Smykla R Lee FY Houghton PJ In vivo evaluation of ixabepilone (BMS247550), a novel epothilone B derivative, against pediatric cancer models Clin Cancer Res 2005 11 6950 6958 10.1158/1078-0432.CCR-05-0740 16203787

[B13] Houghton PJ Morton CL Tucker C Payne D Favours E Cole C Gorlick R Kolb EA Zhang W Lock R The pediatric preclinical testing program: description of models and early testing results Pediatr Blood Cancer 2007 49 928 940 10.1002/pbc.21078 17066459

[B14] Meyer WH Houghton JA Houghton PJ Webber BL Douglass EC Look AT Development and characterization of pediatric osteosarcoma xenografts Cancer Res 1990 50 2781 2785 2328504

[B15] Mischek D Steinborn R Petznek H Bichler C Zatloukal K Sturzl M Gunzburg WH Hohenadl C Molecularly characterised xenograft tumour mouse models: valuable tools for evaluation of new therapeutic strategies for secondary liver cancers J Biomed Biotechnol 2009 2009 437284 19300524 10.1155/2009/437284 PMC2655652

[B16] Rubio-Viqueira B Jimeno A Cusatis G Zhang X Iacobuzio-Donahue C Karikari C Shi C Danenberg K Danenberg PV Kuramochi H An in vivo platform for translational drug development in pancreatic cancer Clin Cancer Res 2006 12 4652 4661 10.1158/1078-0432.CCR-06-0113 16899615

[B17] Neale G Su X Morton CL Phelps D Gorlick R Lock RB Reynolds CP Maris JM Friedman HS Dome J Molecular characterization of the pediatric preclinical testing panel Clin Cancer Res 2008 14 4572 4583 10.1158/1078-0432.CCR-07-5090 18628472 PMC4209898

[B18] Whiteford CC Bilke S Greer BT Chen Q Braunschweig TA Cenacchi N Wei JS Smith MA Houghton P Morton C Credentialing preclinical pediatric xenograft models using gene expression and tissue microarray analysis Cancer Res 2007 67 32 40 10.1158/0008-5472.CAN-06-0610 17210681

[B19] Jimeno A Feldmann G Suarez-Gauthier A Rasheed Z Solomon A Zou GM Rubio-Viqueira B Garcia-Garcia E Lopez-Rios F Matsui W A direct pancreatic cancer xenograft model as a platform for cancer stem cell therapeutic development Mol Cancer Ther 2009 8 310 314 10.1158/1535-7163.MCT-08-0924 19174553 PMC3033101

[B20] Pearce M Culinan A Hogg G Hosseni D Ehrich M Mutation profiling in tumor samples using the Sequenom OncoCarta™ Panel Nature Methods 2009 6 vii viii

[B21] Thomas RK Baker AC Debiasi RM Winckler W Laframboise T Lin WM Wang M Feng W Zander T MacConaill L High-throughput oncogene mutation profiling in human cancer Nat Genet 2007 39 347 351 10.1038/ng1975 17293865

[B22] Nikolsky Y Kirillov E Zuev R Rakhmatulin E Nikolskaya T Functional analysis of OMICs data and small molecule compounds in an integrated "knowledge-based" platform Methods Mol Biol 2009 563 177 196 10.1007/978-1-60761-175-2_10 19597786

[B23] Zheng Q Wang XJ GOEAST: a web-based software toolkit for Gene Ontology enrichment analysis Nucleic Acids Res 2008 36 W358 W363 10.1093/nar/gkn276 18487275 PMC2447756

[B24] Fiebig HH Neumann HA Henss H Koch H Kaiser D Arnold H Development of three human small cell lung cancer models in nude mice Recent Results Cancer Res 1985 97 77 86 10.1007/978-3-642-82372-5_8 2986247

[B25] Houghton JA Williams LG Torrance PM Houghton PJ Determinants of intrinsic sensitivity to Vinca alkaloids in xenografts of pediatric rhabdomyosarcomas Cancer Res 1984 44 582 590 6692363

[B26] Thompson J George EO Poquette CA Cheshire PJ Richmond LB de Graaf SS Ma M Stewart CF Houghton PJ Synergy of topotecan in combination with vincristine for treatment of pediatric solid tumor xenografts Clin Cancer Res 1999 5 3617 3631 10589779

[B27] Dobrolecki LE Landis MD Zhang X Huang J Lai Q Wong H Contreras A Vahdat LD Lewis MT CCJ Preclinical utility of xenografted human breast cancer tumor models Keystone Symposia-Stem Cells, Cancer and Metastasis; March 6–11, 2011 2011 Keystone, CO, Silverthorne, CO

[B28] Zorzoli A Di Carlo E Cocco C Ognio E Ribatti D Ferretti E Dufour C Locatelli F Montagna D Airoldi I Interleukin-27 inhibits the growth of pediatric acute myeloid leukemia in NOD/SCID/Il2rg−/− mice Clin Cancer Res 2012 18 1630 1640 10.1158/1078-0432.CCR-11-2432 22383738

[B29] Malaise M Neumeier M Botteron C Dohner K Reinhardt D Schlegelberger B Gohring G Gruhn B Debatin KM Corbacioglu S Stable and reproducible engraftment of primary adult and pediatric acute myeloid leukemia in NSG mice Leukemia 2011 25 1635 1639 10.1038/leu.2011.121 21647161

[B30] Garber K Personal mouse colonies give hope for pancreatic cancer patients J Natl Cancer Inst 2007 99 105 107 10.1093/jnci/djk046 17227991

[B31] Vilardell F Iacobuzio-Donahue CA Cancer gene profiling in pancreatic cancer Methods Mol Biol 2010 576 279 292 19882267 10.1007/978-1-59745-545-9_14

[B32] Harris TJ McCormick F The molecular pathology of cancer Nat Rev Clin Oncol 2010 7 251 265 10.1038/nrclinonc.2010.41 20351699

[B33] Irahara N Baba Y Nosho K Shima K Yan L Dias-Santagata D Iafrate AJ Fuchs CS Haigis KM Ogino S NRAS mutations are rare in colorectal cancer Diagn Mol Pathol 2010 19 157 163 10.1097/PDM.0b013e3181c93fd1 20736745 PMC2929976

[B34] Shepherd C Puzanov I Sosman JA B-RAF inhibitors: an evolving role in the therapy of malignant melanoma Curr Oncol Rep 2010 12 146 152 10.1007/s11912-010-0095-2 20425073

[B35] Hu T Li C Convergence between Wnt-beta-catenin and EGFR signaling in cancer Mol Cancer 2010 9 236 20828404 10.1186/1476-4598-9-236 PMC2944186

[B36] Ott PA Adams S Small-molecule protein kinase inhibitors and their effects on the immune system: implications for cancer treatment Immunotherapy 2011 3 213 227 10.2217/imt.10.99 21322760 PMC4009988

[B37] Singh RR Cho-Vega JH Davuluri Y Ma S Kasbidi F Milito C Lennon PA Drakos E Medeiros LJ Luthra R Vega F Sonic hedgehog signaling pathway is activated in ALK-positive anaplastic large cell lymphoma Cancer Res 2009 69 2550 2558 10.1158/0008-5472.CAN-08-1808 19244133

[B38] Nisolle M Casanas-Roux F Marbaix E Jadoul P Donnez J Transplantation of cultured explants of human endometrium into nude mice Hum Reprod 2000 15 572 577 10.1093/humrep/15.3.572 10686198

[B39] Shimoda M Chen S Noguchi H Matsumoto S Grayburn PA In vivo non-viral gene delivery of human vascular endothelial growth factor improves revascularisation and restoration of euglycaemia after human islet transplantation into mouse liver Diabetologia 2010 53 1669 1679 10.1007/s00125-010-1745-5 20405100 PMC3804430

[B40] Abir R Fisch B Jessel S Felz C Ben-Haroush A Orvieto R Improving posttransplantation survival of human ovarian tissue by treating the host and graft Fertil Steril 2011 95 1205 1210 10.1016/j.fertnstert.2010.07.1082 20817170

[B41] Pelleitier M Montplaisir S The nude mouse: a model of deficient T-cell function Methods Achiev Exp Pathol 1975 7 149 166 1105061

[B42] Kanwar JR Berg RW Yang Y Kanwar RK Ching LM Sun X Krissansen GW Requirements for ICAM-1 immunogene therapy of lymphoma Cancer Gene Ther 2003 10 468 476 10.1038/sj.cgt.7700590 12768192

[B43] Stewart CA Vivier E Kitamura D Strategies of Natural Killer (NK) Cell Recognition and Their Roles in Tumor Immunosurveillance How the Immune System Recognizes Self and Nonself Volume 2008 Springer, New York 37 81

[B44] Ohyama C Kanto S Kato K Nakano O Arai Y Kato T Chen S Fukuda MN Fukuda M Natural killer cells attack tumor cells expressing high levels of sialyl Lewis x oligosaccharides Proc Natl Acad Sci U S A 2002 99 13789 13794 10.1073/pnas.212456599 12370411 PMC129776

[B45] Ma D Luyten GP Luider TM Niederkorn JY Relationship between natural killer cell susceptibility and metastasis of human uveal melanoma cells in a murine model Invest Ophthalmol Vis Sci 1995 36 435 441 7843912

[B46] Hansson M Bakacs KK Kiessling R Klein G Intra- and interspecies reactivity of human and mouse natural killer (NK) cells J Immunol 1978 121 6 12 78947

[B47] Nieswandt B Hafner M Echtenacher B Mannel DN Lysis of tumor cells by natural killer cells in mice is impeded by platelets Cancer Res 1999 59 1295 1300 10096562

[B48] Welsh RM Mouse natural killer cells: induction specificity, and function J Immunol 1978 121 1631 1635 361888

[B49] Shouval D Rager-Zisman B Quan P Shafritz DA Bloom BR Reid LM Role in nude mice of interferon and natural killer cells in inhibiting the tumorigenicity of human hepatocellular carcinoma cells infected with hepatitis B virus J Clin Invest 1983 72 707 717 10.1172/JCI111020 6192149 PMC1129230

[B50] Carroll JL Nielsen LL Pruett SB Mathis JM The role of natural killer cells in adenovirus-mediated p53 gene therapy Mol Cancer Ther 2001 1 49 60 12467238

